# Vasculogenic and angiogenic potential of adipose stromal vascular fraction cell populations in vitro

**DOI:** 10.1007/s11626-017-0213-7

**Published:** 2017-12-01

**Authors:** Joseph S. Zakhari, Jacob Zabonick, Brian Gettler, Stuart K. Williams

**Affiliations:** Bioficial Organs Program, Cardiovascular Innovation Institute, 302 E. Muhammad Ali Blvd, Louisville, KY 40202 USA

**Keywords:** Angiogenesis, Vasculogenesis, Adipose, Stromal vascular fraction

## Abstract

Adipose-derived stromal vascular fraction (SVF) is a heterogeneous cell source that contains endothelial cells, pericytes, smooth muscle cells, stem cells, and other accessory immune and stromal cells. The SVF cell population has been shown to support vasculogenesis in vitro as well vascular maturation in vivo. Matrigel, an extracellular matrix (ECM) mixture has been utilized in vitro to evaluate tube formation of purified endothelial cell systems. We have developed an in vitro system that utilizes freshly isolated SVF and ECM molecules both in pure form (fibrin, laminin, collagen) as well as premixed form (Matrigel) to evaluate endothelial tip cell formation, endothelial stalk elongation, and early stages of branching and inosculation. Freshly isolated SVF rat demonstrate cell aggregation and clustering (presumptive vasculogenesis) on Matrigel ECM within the first 36 h of seeding followed by tip cell formation, stalk cell formation, branching, and inosculation (presumptive angiogenesis) during the subsequent 4 days of culture. Purified ECM molecules (laminin, fibrin, and collagen) promote cell proliferation but do not recapitulate events seen on Matrigel. We have created an in vitro system that provides a functional assay to study the mechanisms of vasculogenesis and angiogenesis in freshly isolated SVF to characterize SVF’s blood vessel forming potential prior to clinical implantation.

**Electronic supplementary material:**

The online version of this article (10.1007/s11626-017-0213-7) contains supplementary material, which is available to authorized users.

## Introduction

Tissue engineering, the microvasculature is a novel approach towards therapeutic reperfusion for ischemic disease states such as peripheral vascular disease and acute myocardial infarction (Laschke and Menger 2015; Laschke and Menger 2016; Riemenschneider et al. 2016; Sun et al. 2016; Bogorad et al. 2017; Kc et al. 2017; Valarmathi et al. 2017). Microvascular engineering requires both cellular and extracellular components to recapitulate the microenvironment and tissue organization of endogenous microcirculation. Indeed, many researchers have isolated vascular components from a variety of tissues including endothelial-only populations from both large vessel (human umbilical vein and other large adult vessels) and microvascular tissues (Haug et al. 2015; Sasagawa et al. 2016; Morrissette-McAlmon et al. 2017). In vitro studies have established the ability of these endothelial cells to undergo the formation of tube-like structures, a process that is highly dependent on the extracellular matrix used as a substrate (Madri and Williams 1983).

Additionally, adipose-derived stromal vascular fraction (SVF) cells have been proposed as a source of cells for microvascular tissue engineering (Leblanc et al. 2013; Nunes et al. 2013; Maijub et al. 2015; Jin et al. 2017). Adipose-derived SVF represents a heterogeneous cell population (Wagner et al. 1972; Wagner and Matthews 1975) that has demonstrated efficacy in the formation of functional microcirculation following implantation (Shepherd et al. 2007; Hiscox et al. 2008; Chang et al. 2012; Leblanc et al. 2012). These in vivo studies are based, in part, on the observed ability of SVF-derived, cultured, microvascular endothelial cells to undergo tube formation in vitro (Madri and Williams 1983); however, and the impetus for the studies reported here, the in vitro formation of tube-like structures by freshly isolated, heterogeneous SVF cell populations has not been reported.

Previous in vitro “angiogenesis” assays have utilized endothelial cell cultures placed on collagen type IV or Matrigel-treated culture surfaces with a temporal sequence of tube-like structure formation that occurs within 24 h of plating (Madri and Williams 1983; Nicosia and Ottinetti 1990; Albini 2016; Brown et al. 2016). It can be argued that this self-assembly of cells into tube-like structures does not recapitulate the process known as angiogenesis (i.e., formation of new vessels from pre-existing vessel) and recapitulates only one part of the vasculogenic process (i.e., formation of blood vessels from cellular components). The current studies are based on preliminary observations that freshly isolated adipose-derived SVF cell populations that do not undergo tube-like structure formation in the first 24 h after plating on Matrigel. However, extending the assessment of the adipose SVF cell populations plated on Matrigel for a 7-d period resulted in the observation of cell aggregation, tip cell formation, sprouting of vascular structures followed by branching, and inosculation. Herein, we describe an in vitro assay that we propose which captures each step of vasculogenesis and angiogenesis over a 7-d period via time-lapse microscopy utilizing rat epididymal fat or human lipoaspirate-derived SVF as a cell source.

## Materials and Methods

### **SVF isolation from Sprague Dawley rats**

Rat epididymal fat pads were excised from 6- to 8-month-old Sprague Dawley rats at weights greater than 250 g under sterile surgical procedure and isoflurane anesthesia. All procedures were reviewed and approved under the University of Louisville’s Institutional Animal Care and Use Committee. Excised fat pads were placed in PBS containing 0.1% bovine serum albumin (Sigma-Aldrich, St. Louis, MO) and kept at 4°C for 15 min prior to digestion. Samples were washed with BSA-PBS and minced for 2 min until particulates could pass through a 50-mL aspirating pipette. Two milligrams of type I collagenase (Worthington Biochemical Corporation, Lakewood, NJ) was added per milliliter of fat, aliquoted at 20 mL total volume in 50 mL conicle centrifuge tubes and rotated in an Enviro-Genie incubator (Scientific Industries, Bohemia, NY) at 35 rpm and 37°C for 35 min. Samples were pelleted via centrifugation at 350×*g* for 4 min at room temperature (RT). Buoyant adipocytes were aspirated and discarded, and dense cellular pellets were suspended and washed one time in BSA-PBS. Samples were recentrifuged for 4 min at 350×*g*. The SVF pellet was filtered through a 250-μm mesh filter (Tissue Genesis Incorporated, Honolulu, HI) and collected into DMEM containing endothelial cell growth supplement, 2 mM l-glutamine, 10% fetal bovine serum, and 5 mM Hepes buffer. Samples were kept at RT prior to plating.

### **SVF isolation from human lipoaspirate**

Human lipoaspirate was digested under sterile conditions following the same protocol as rat SVF isolation. Briefly, lipoaspirate was provided from de-identified patients undergoing general non-ultrasound-guided lipoaspiration. Lipoaspirate was washed with 0.1% BSA-PBS to provide non-bloody yellow fat. Subsequently, Worthington collagenase type I enzyme was added to obtain a final concentration of 6 mg/mL collagenase to fat volume. Samples were digested in an Enviro-Genie at 35 rpm and 37°C for 35 min. Samples were pelleted via centrifugation at 350×*g* for 4 min at RT. Buoyant adipocytes were aspirated and discarded, and dense cellular pellets were suspended and washed one time in BSA-PBS. Samples were recentrifuged for 4 min at 350×*g*. The SVF pellet was filtered through a 250-μm mesh filter. The SVF pellet was resuspended in M199 media containing endothelial cell growth supplement, 10% fetal bovine serum, 2 mM l-glutamine, and 5 mM Hepes buffer.

### **Angiogenesis assay and image capture**

Either 500 μL of growth factor-reduced Matrigel (Corning, Corning, NY), 5 mg/mL bovine fibrin (Sigma, St. Louis, MO), 3 mg/mL rat tail collagen (Corning, Corning, NY), 1% porcine gelatin (Sigma, St. Louis, MO), or 2 μg Laminin 332 (Abcam, Cambridge, MA) was added to each well of a 48-well polystyrene cell culture plate (Corning, Corning, NY) at 4°C. Fibrin was polymerized through thrombin activation at 2 U/mg fibrinogen. Collagen was polymerized after the addition of 4 N NaOH to a pH of 7.7 and incubation at 37°C. The Matrigel, fibrin, and collagen hydrogels solidified after 15 min of incubation at 37°C. 1.6 × 10^5^ SVF cells were subsequently plated per well and allowed to adhere to the ECM overnight in a tissue culture incubator (37°C, 5% CO_2_). Media was changed the following d, and the 48-well plates were loaded into a Cytation 5 cell imaging multi-mode reader (Biotek, Winooski, VT) set to 37°C and 5% CO_2_. A time-lapse capture experiment was created in Gen5 software utilizing a ×4 objective capturing phase contrast images at 15 min intervals with an endpoint of 160 h*.* The media was changed every other day. Specific inhibitors of angiogenesis were added at 25 or 1 μM at each media change over the 160-h incubation time. Inhibitors included imatinib mesylate (1 μM) (Sigma-Aldrich, St. Louis, MO); DAPT (25 μM) (Abcam, Cambridge, UK); ZM 306416 (25 μM) (Selleckchem, Houston, TX); and ATN 161 (25 μM) (Peptides International, Louisville, KY).

Image files were stitched together using ImageJ software. Individual still images were selected at remarkable time points to demonstrate events such as clustering (18 h), tip cell formation (36 h), stalk cell formation (60 h), and inosculation (112 h). SVF and huvec cells grown on Matrigel to the 112-h endpoint were fixed with 4% paraformaldehyde for 15 min at RT, permeabilized with 0.1% Triton X 100 for 15 min at RT, and stained with *Griffonia simplicifolia* 1 lectin conjugated to FITC (1:500) (Vector Biotechnologies, Burlingame, CA) and α-smooth muscle actin mouse monoclonal primary antibody (1:500) (Santa Cruz Biotechnology, Dallas, TX) overnight at 4°C to visualize endothelial and smooth muscle cells, respectively.

### **Event counting and statistical analysis**

Cell clusters were automatically counted using Gen5 software under cellular analysis tools with threshold intensities set at values less than 10,000, minimum object size set at 25 μm and maximum object size set at 1 mm. These counts along with manual counts of tip cells and stalk cells were taken using still frames at 18, 36, 60, and 112 h, respectively, of both control and treated groups. Graphs and statistics were run with GraphPad Prism v.7 Software (La Jolla, CA). *P* values were calculated via one-way ANOVAs with means and standard deviations plotted per group as compared to vehicle only (control group).

## Results

**Figure 1.**
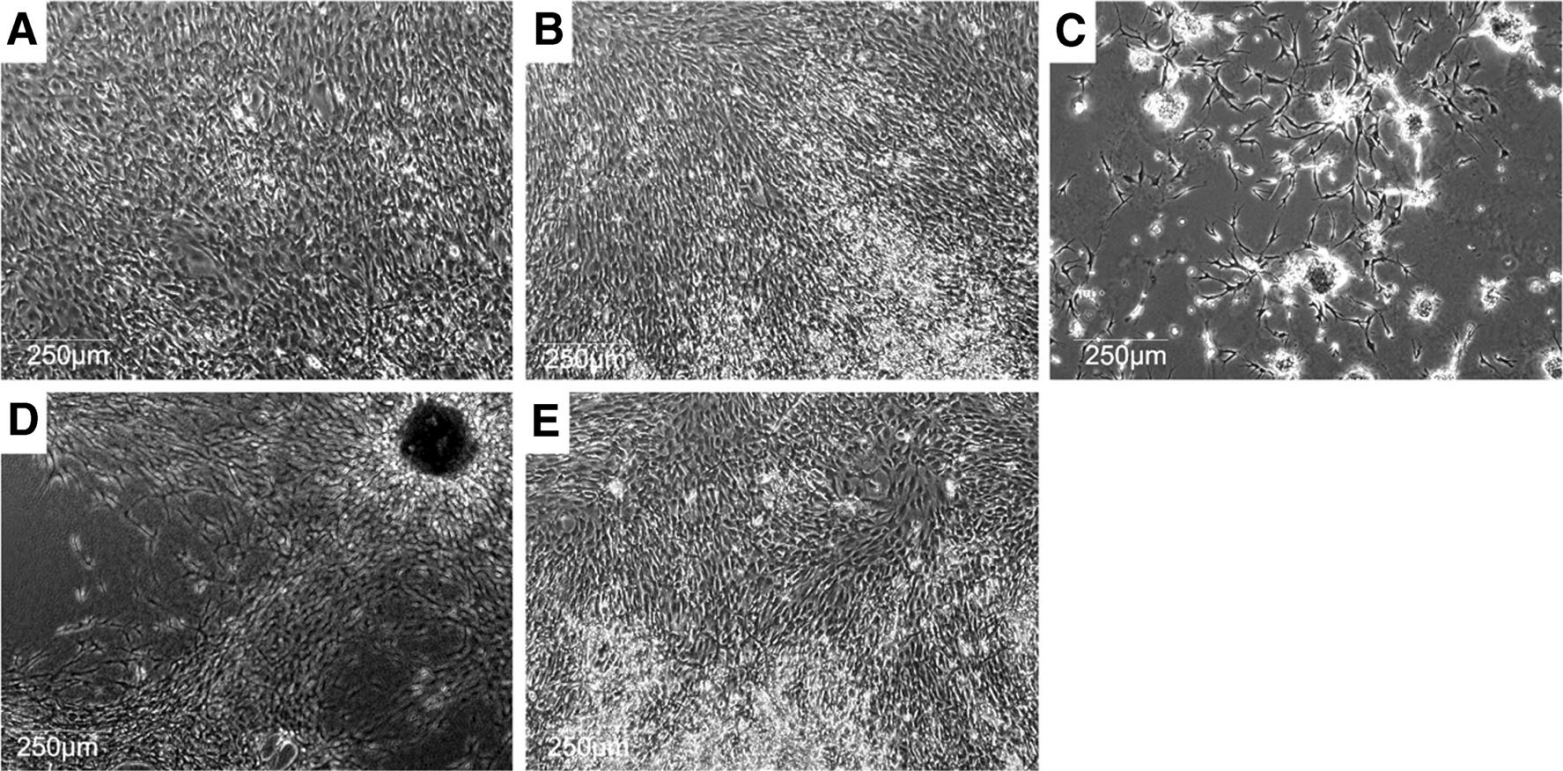
×4 phase contrast images of SVF grown on 3 mg/mL collagen I (*A*), 1% gelatin (*B*), Matrigel (*C*), 5 mg/mL fibrin (*D*), or 2 μg laminin 332 (*E*) after 112 h of incubation. Monolayers of cells are formed on collagen I (*A*), gelatin (*B*) and laminin 332 (*E*) with cell clustering demonstrated on Matrigel (*C*) and fibrin (*D*). SVF forms microvascular networks on Matrigel (*C*).

**Figure 2.**
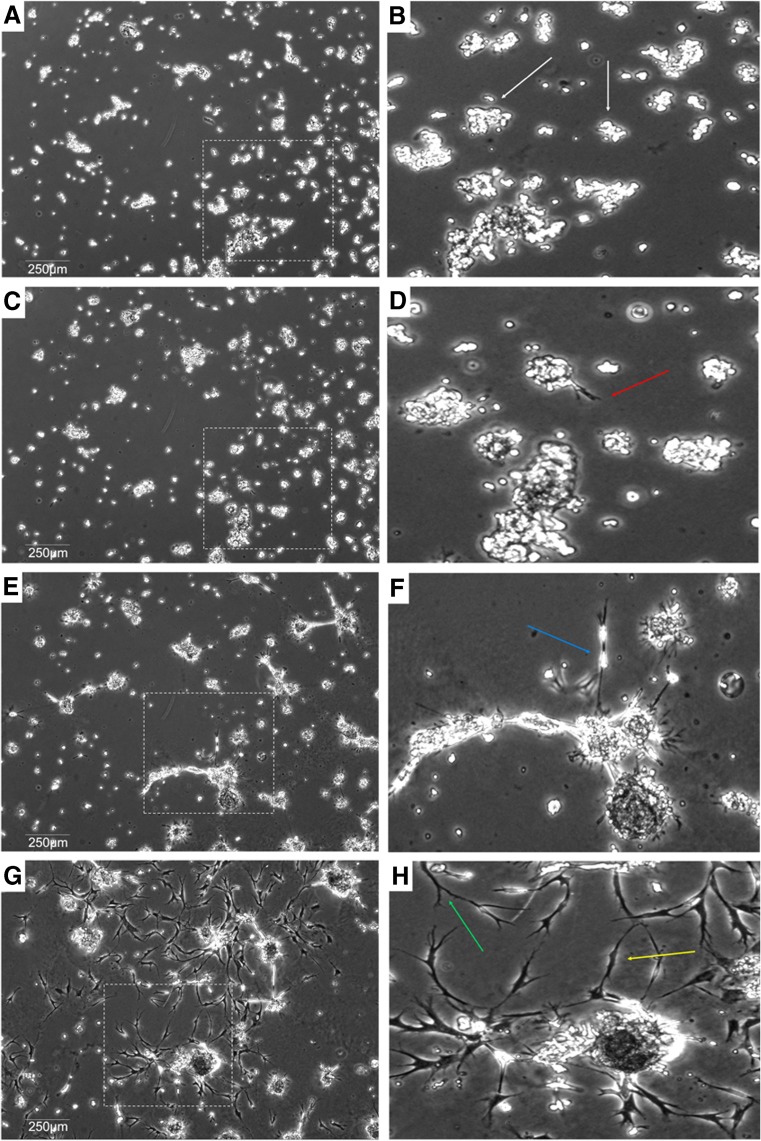
×4 phase contrast images of SVF grown on Matrigel at 18 h (*A*, *B*); 36 h (*C*, *D*); 60 h (*E*, *F*); and 112 h (*G*, *H*). Images *B*, *D*, *F*, and H are ×3 optical zooms of *boxed sections* of images *A*, *C*, *E*, and *G*, respectively. *White arrows* demonstrate initial cell clustering occurring at 18 h. Tip cell formation occurs at 36 h as marked by the *red arrow*. Stalk cell formation and elongation occurs at 60 h as marked by the *blue arrow*. Branching (*green arrow*) and inosculation (*yellow arrow*) can be seen at 118 h in vitro.

SVF grown on fibrin, collagen, gelatin, and laminin extracellular matrices behave differently than SVF on Matrigel. Cells form monolayers on the aforementioned ECM, whereas SVF grown on Matrigel undergoes phenotypic change resembling vasculogenesis and angiogenesis after 112 h (Fig. **1**). Subpopulations of SVF cells plated on Matrigel begin to migrate and form clusters ranging in size and cell number after 18 h of incubation (Fig. **2** *A*, *B*). These clustering events resemble angioblasts forming blood islands, the initial step s of vasculogenesis in the embryo. The next phenomenon occurs when tip cells subsequently migrate away from the cluster around 36 h of incubation (Fig. **2** *C*, *D*). During angiogenesis, endothelial cells emerge from a pre-existing vessel and remodel surrounding ECM via matrix metalloprotease secretion. Ultimately, these tip cells migrate away from the nascent vessel along with additional endothelial cells creating collateral vessels that will form lumens over time. Tip cells must have additional endothelial cells that migrate behind them to properly signal growth and proliferation events away from the nascent vessel. These specific endothelial cells are termed stalk cells in angiogenesis.

Indeed, after tip cell migration, we see the development of stalk cells growing out of the cluster at 60 h of incubation as demonstrated in Fig. **2** *E*, *F*. Stalk cells signal adjacent tip cells through the notch pathway to maintain tip phenotype as well as maintain stalk phenotype. Briefly, VEGF-A signaling through VEGFR-2 causes an upregulation of delta-like ligand 4 which subsequently binds notch to facilitate receptor cleavage by γ-secretase. Soluble notch can then translocate to the nucleus and cause downstream transcriptional activation; including the upregulation of VEGFR-1, a receptor with low signaling activity via the VEGF-A ligand. VEGFR-2 is downregulated while VEGFR-1 is upregulated on stalk cells, promoting the stalk cell phenotype.

**Figure 3.**
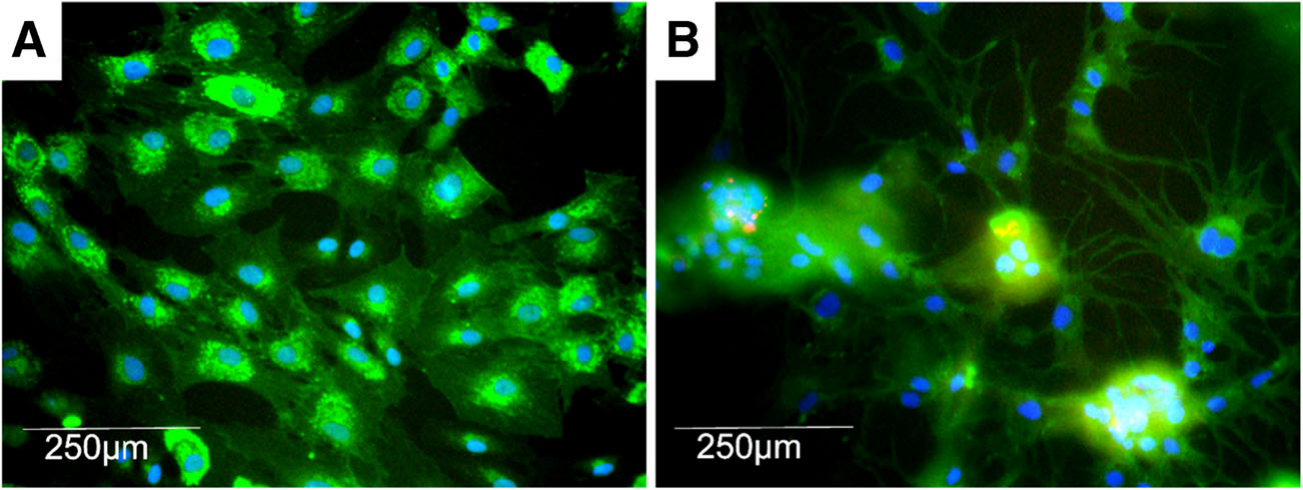
×10 fluorescent images of SVF grown on 1% gelatin (*A*) or Matrigel (*B*) for 112 h. Blue: DAPI, green: *Griffonia simplicifolia* 1 endothelial specific lectin-FITC, and red: α smooth muscle actin-rhodamine.

After 60 h of incubation and up to the endpoint of 112 h, we see the retention of stalk cells as well as tip cell migration and ultimately increased vessel density and complexity including branch points and inosculation events as demonstrated in Fig. **2** *G*, *H*. To validate that the neovasculature was comprised primarily of the endothelial cell population within SVF, cultures incubated to 112 h were stained with *Griffonia simplicifolia* 1, an isolectin that binds endothelial cell-specific glycoproteins and is widely used in the field as a rat endothelial cell marker. Figure **3** demonstrates that both on gelatin and Matrigel there is a predominance of endothelial cells; however, only on Matrigel do GS1-positive tip cell and stalk cell phenotypes form.

**Figure 4.**
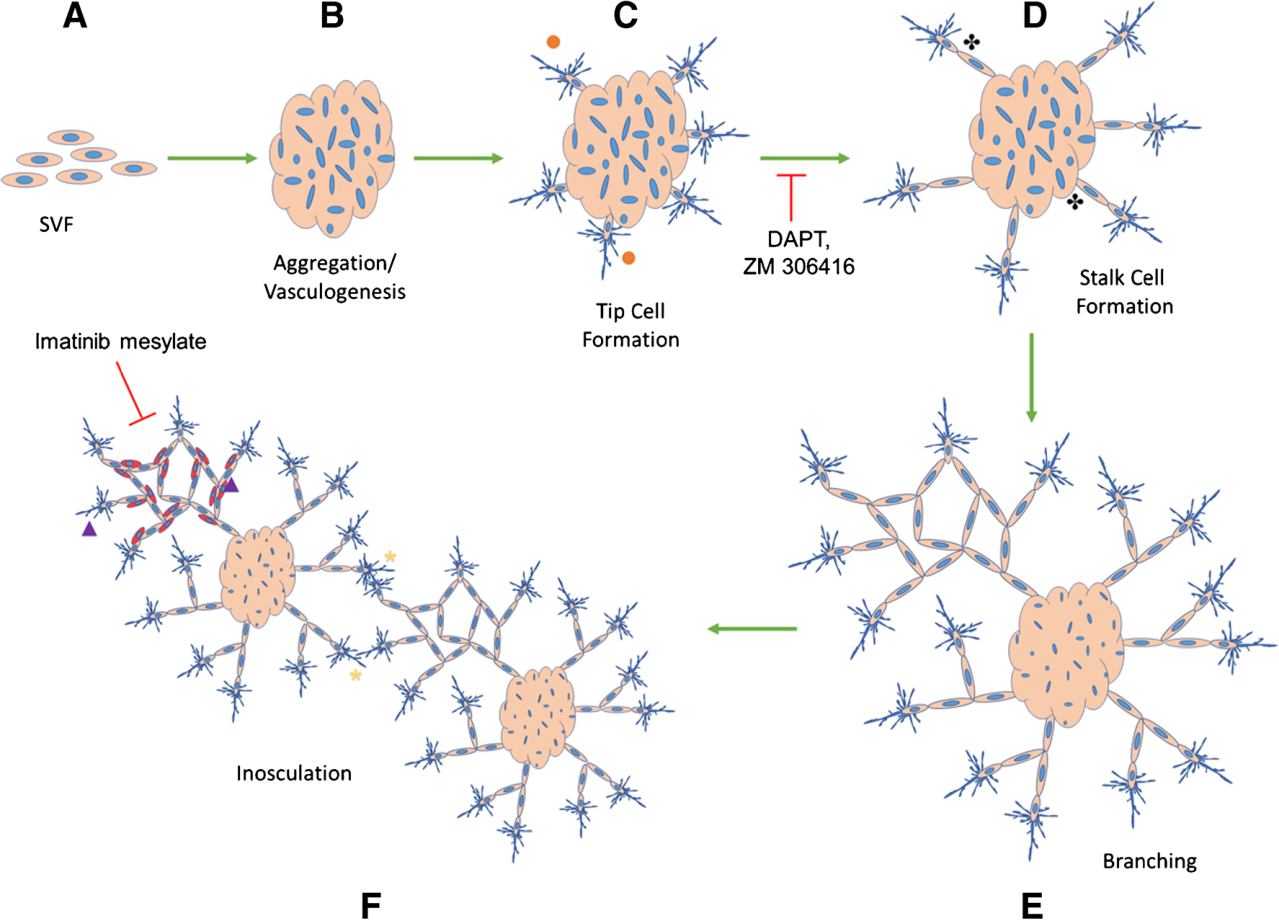
Schematic of SVF microvascular assembly on Matrigel in vitro. Digested SVF cells (*A*) self-assemble into a cluster of cells by 18 h (*B*). By 36 h, endothelial cells sprout out of the cluster with dendritic-like extensions (tip cells: orange ●) (*C*). DAPT, an inhibitor of the γ-secretase that cleaves notch to its soluble ligand, blocks tip cell signaling to stalk cells, inhibiting stalk cell formation. Stalk cell formation occurs when notch signaling leads to a higher expression of VEGR-1 on the adjacent cell. ZM 306416 blocks VEGFR-1 and thus, stalk cell phenotype. In the absence of inhibitors, stalk cells (black ) migrate away from clusters behind tip cells by 60 h (*D*). Microvascular networks continue to grow after 112 h and demonstrate increased complexity (*E*). Networks from adjacent clusters inosculate with one another (yellow *) and recruit perivascular support cells to endothelial tubes (purple ▲) (*F*). PDGFR-B is found on perivascular support cells and activated by endothelial PDGF-β, which signals pericyte recruitment and tube stabilization. Imatinib mesylate inhibits PDGFR-B.

To determine if SVF was undergoing vasculogenesis and angiogenesis specifically, the assay was repeated in the presence of the inhibitors DAPT, ZM 306416, imatinib mesylate, and ATN-161. DAPT is an inhibitor of the γ-secretase that cleaves notch receptor to its soluble ligand preventing stalk cell phenotype. ZM 306416 is a small molecule inhibitor of VEGFR-1 (Antczak et al. 2012), again inhibiting stalk cell phenotype via the inhibition of VEGFR-1. Imatinib mesylate blocks platelet-derived growth factor receptor-B (PDGFR-B) (Heymach et al. 2004) which is found on mural cells and instrumental in the recruitment of perivascular support cells to developing endothelial tubes necessary for stabilization. ATN-161 is a small molecule inhibitor of the α_5_β_1_ integrin (Nourse et al. 2009), a membrane-bound protein that binds ECM such as fibrin, fibronectin, and vitronectin (Fig. 4). During the remarkable time points (18, 36, 60, and 112 h), the data on cluster number, the cluster size, the percentage of clusters with tip cells, and the percentage of clusters with stalk cells were collected in the presence of each inhibitor and compared to DMSO (vehicle only) control via one-way ANOVA.

**Figure 5.**
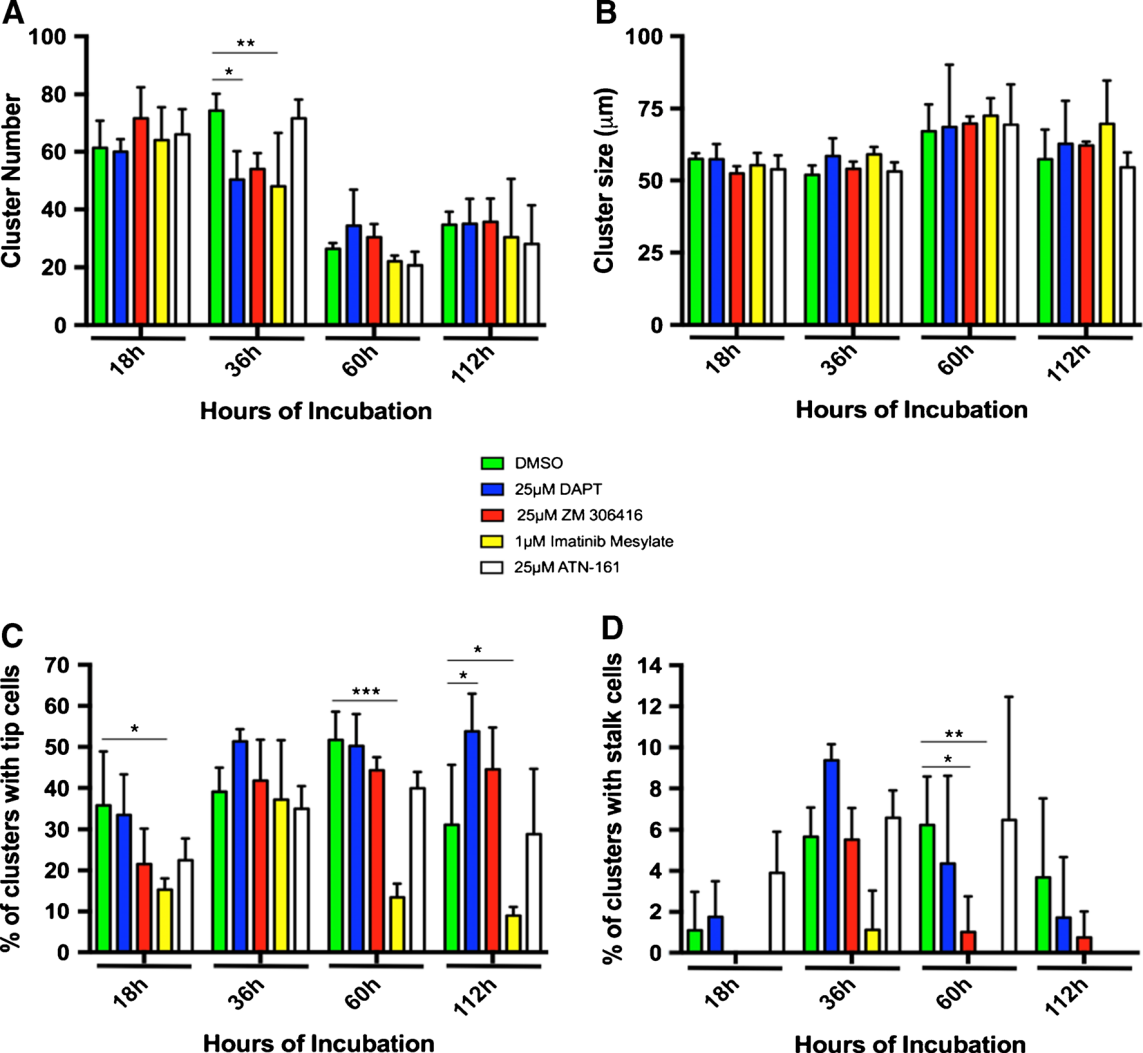
Number of SVF cell clusters (grouping of two or more cells over 25 μm in total size) at 18, 36, 60, and 112 h of incubation in the presence of inhibitors (*A*). Cluster size (micrometer) at 18, 36, 60, and 112 h of incubation in the presence of inhibitors (*B*). Percentage of clusters containing tip cells at 18, 36, 60, and 112 h of incubation in the presence of inhibitors (*C*). Percentage of clusters containing stalk cells at 18, 36, 60, and 112 h of incubation in the presence of inhibitors (*D*). Angiogenic inhibitors (in DMSO) were added at days 0, 2, 4, and 6. *p* < 0.05 *, *p* < 0.005 **, *p* < 0.0005 ***.

Cluster number was significantly reduced in the presence of 25-μM DAPT as well as 1-μM imatinib mesylate at 36 h as compared to DMSO controls (*p* < 0.05, *p* < 0.005, respectively, Fig. 5 *A*). It is possible that the biochemical pathways involved in stalk cell formation as well as mural cell migration may play a role in the recruitment of neighboring SVF cells during initial cell aggregation. In addition, a decreasing trend in overall cluster number over time is noted, which can be explained by the neighboring clusters grouping together forming larger units, decreasing total counts. This phenomenon may be due to a variety of cell-cell signaling promoting cell migration and interaction leading to myofibroblastic contraction pulling groupings together (Supplemental Figure 1). Indeed, myofibroblasts as well as other contractile cells have been found in adipose-derived SVF (Diaz-Flores et al. 2015). Cluster size does not seem to change between inhibitor and non-inhibitor groups; however, there is an increased trend in average size of clusters over 112 h across all groups (Fig. 5 *B*).

While tip cell phenotype initially begins around 16 h of incubation, certain clusters retain tip cells even up until 112 h of incubation. There is a significant reduction in tip cell expression in the presence of imatinib mesylate at 18 h of incubation (*p* < 0.05) with a non-significant decrease in tip cells in the presence of the other angiogenic inhibitors. Again, at 60 h of incubation, there is a significant decrease in tip cells in the presence of imatinib mesylate (*p* < 0.0005).

As tip cells migrate, stalk cells form to stabilize and facilitate tip cell migration away from clusters, up until tip cells inosculate with other microvessels, forming a neovascular network. These events occur at 112 h, where control groups begin to decrease tip cell expression because of inosculation events, forming continuous vessel networks. However, cells treated with DAPT have statistically higher amounts of tip cells as compared to DMSO (*p* < 0.05). This retention of tip cells may be attributable to the loss of stalk cell production via notch pathway inhibition. Tip cells still can form and begin initial migration away from clusters, but cannot migrate more than a cell length away due to the lack of stalk cell production. Tip cell percentage at 112 h in the presence of imatinib mesylate is also significantly lower than the vehicle only group (*p* < 0.05, Fig. 5 *C*). These data are corroborated with stalk cell counts at the significant time points. Indeed, while tip cell percentage remains high in the presence of DAPT, and ZM 306416, stalk cell percentages decrease significantly in the presence of ZM 306416 and imatinib mesylate at 60 h of incubation (Fig. 5 *D*).

## Discussion

The formation of blood vessels in vivo occurs due to the processes of vasculogenesis during development and angiogenesis during development, pathologic, and physiologic conditions. The study of vasculogenesis and angiogenesis have been aided by in vitro models that have utilized the ability to culture expand endothelial cells and establish conditions that support the development of endothelial tube-like structures in 2D culture (Madri and Williams 1983; Staton et al. 2009; Arnaoutova and Kleinman 2010). However, an in vitro model that recapitulates all vasculogenic and angiogenic processes has not been established.

The formation of tube-like structures by endothelial cells plated onto extracellular matrix has often been described as a model of angiogenesis in vitro but arguably this is not accurate since only one of the processes necessary for angiogenesis occurs—tube formation. The results presented support the conclusion that adipose-derived stromal vascular fraction cell populations plated onto Matrigel, immediately after isolation, exhibit both vasculogenic and angiogenic elements of microvessel formation.

The self-assembly of cells into microvessels requires the appropriate cells to communicate with one another via paracrine and mechanical factors to promote proliferation, migration, and inosculation, ultimately forming functional microcirculation from individualized cellular components (Lin et al. 2016). Recapitulating this process in vitro requires the presence of each cell type that makes up functional microcirculation, including but not limited to endothelial cells, smooth muscle cells, pericytes and tissue resident immune cells (Mildmay-White and Khan 2016). Adipose-derived SVF populations undergo vasculogenesis and angiogenesis as demonstrated by events such as cell clustering, followed by tip cell formation, stalk cell formation, branching, and ultimately complex network formation including the inosculation of adjacent vessel projections.

These events can be validated by well-known inhibitors of angiogenesis, some commonly used in the clinic as antiangiogenic tumor pharmacotherapies. In the literature as well as in our assay, DAPT, an inhibitor of the γ-secretase that cleaves NOTCH to its soluble ligand, prevents endothelial stalk cell formation and subsequent tip cell migration (Tung et al. 2012; Blanco and Gerhardt 2013). While the notch pathway is not endothelial-specific only, it provides an important bottleneck in the promotion of the tip or stalk cell phenotype in endothelial cells. It is highly conserved in many cell types establishing polarity. ZM 306416, a small molecule competitive inhibitor of VEGFR-1 inhibits stalk cell phenotype. Additionally, imatinib mesylate, an inhibitor of PDGFR-B, a receptor found on mural and perivascular support cells, prevents their recruitment to new endothelial tubes via PDGF-β ligand chemotaxis. This PDGF axis is instrumental in the mobilization and recruitment of stabilizing cells during angiogenesis, which ultimately leads to retention of non-leaky, functional neovasculature (Gaengel et al. 2009). These inhibitors validate the vasculogenic and angiogenic processes occurring in this assay and were chosen based from well-known mechanisms of angiogenesis. However, the assay provides the opportunity to validate and test a variety of proangiogenic or antiangiogenic pharmacotherapies as well as test cytokines including but not limited to fibroblast growth factor 2, hepatocyte growth factor, ephrin B1, and others in order to assess additional angiogenic pathways.

Indeed, endothelial cell and mural cell self-assembly and proliferation are required to form functional microvasculature; however, extracellular matrix (ECM) components are necessary to stimulate cell growth and maturation (Moore et al. 2015; Fercana et al. 2017). Fully mature vascular networks restructure surrounding ECM, via matrix metalloproteases, to create a distinct basement membrane composed predominantly of type IV collagen and laminins 121 and 332, allowing for appropriate adventitial cell signaling, structural integrity, and decreased permeability (Ebrahem et al. 2010; Sacharidou et al. 2012; Mammoto et al. 2013). Matrigel, a complex mixture of extracellular matrix molecules created from Engelbreth-Holm-Swarm mouse tumor, is an ECM derivative that contains basement membrane proteins necessary to stimulate endothelial cells to migrate, proliferate, and create tube-like structure morphologies. As such, many in vitro angiogenesis assays use Matrigel as the dominant ECM prior to cell plating. It is important to note that the initial vasculogenic and angiogenic events occur within the first 24 h of cell plating on substrates such as Matrigel for pure endothelial cell populations (Hughes 1996). This assay establishes a methodology to evaluate and classify the vasculogenic/angiogenic capability of patient-specific SVF prior to clinical usage.

Although the current study represents a solely in vitro evaluation of vasculogenic and angiogenic processes by adipose-derived SVF cell populations, the discussion of how these results may relate to in vivo vasculogenesis and angiogenesis is warranted. Adipose-derived SVF cell populations are under extensive study for use in clinical applications where tissue revascularization is desired (Carstens et al. 2017). The direct injection of adipose-derived SVF cells into ischemic tissue has been hypothesized to support the formation of new blood vessels in the target tissue. Most studies suggest the action of the SVF is to stimulate new blood vessel formation by angiogenic mechanisms that are by the formation of new vessels from pre-existing vessels. Thus, the SVF cell population is providing a source of paracrine factors. Pre-clinical studies provide an alternative explanation for increased vessel density of SVF-treated ischemic tissue. Using cell markers to evaluate the fate of injected adipose-derived SVF, the cells exhibit the ability to self-assemble into new vessels. The new vessels formed include arterioles, venules, and capillaries, and the cell tracking technology indicates these new vessels originate from the injected SVF cell population (Chang et al. 2012). However, it remains unknown whether the injected SVF exhibits cell aggregation as observed in the current in vitro system. We believe that SVF harbors the appropriate milieu of cells to reform multi-cellular vascular components, including endothelial cells, smooth muscle cells, pericytes, adipose-derived stem cells, and additional immune and stromal cells. The development of this SVF-based automated assay of vasculogenesis may permit the assessment of patient-specific SVF cell populations to analyze vasculogenic and angiogenic potential prior to surgical implantation.

## Electronic supplementary material

Supplemental Figure 1 Time-lapse movie of freshly isolated rat SVF grown on matrigel over 112 h demonstrating aggregation, tip cell formation, stalk cell formation, cellular migration, and vessel network formation. (MOV 90674 kb)
